# Dentifrices, mouthwashes, and remineralization/caries arrestment strategies

**DOI:** 10.1186/1472-6831-6-S1-S9

**Published:** 2006-06-15

**Authors:** Domenick T Zero

**Affiliations:** 1 Indiana University School of Dentistry, Oral Health Research Institute, IN 46202-2876, USA

## Abstract

While our knowledge of the dental caries process and its prevention has greatly advanced over the past fifty years, it is fair to state that the management of this disease at the level of the individual patient remains largely empirical. Recommendations for fluoride use by patients at different levels of caries risk are mainly based on the adage that more is better. There is a general understanding that the fluoride compound, concentration, frequency of use, duration of exposure, and method of delivery can influence fluoride efficacy. Two important factors are (1) the initial interaction of relatively high concentrations of fluoride with the tooth surface and plaque during application and (2) the retention of fluoride in oral fluids after application.

Fluoride dentifrices remain the most widely used method of delivering topical fluoride. The efficacy of this approach in preventing dental caries is beyond dispute. However, the vast majority of currently marketed dentifrice products have not been clinically tested and have met only the minimal requirements of the FDA monograph using mainly laboratory testing and animal caries testing. Daily use of fluoride dental rinses as an adjunct to fluoride dentifrice has been shown to be clinically effective as has biweekly use of higher concentration fluoride rinses. The use of remineralizing agents (other than fluoride), directed at reversing or arresting non-cavitated lesions, remains a promising yet largely unproven strategy. High fluoride concentration compounds, *e.g*., AgF, Ag(NH_3_)_2_F, to arrest more advanced carious lesions with and without prior removal of carious tissue are being used in several countries as part of the Atraumatic Restorative Treatment (ART) approach.

Most of the recent innovations in oral care products have been directed toward making cosmetic marketing claims. There continues to be a need for innovation and collaboration with other scientific disciplines to fully understand and prevent dental caries.

## Introduction

Dental caries remains the most common totally preventable disease facing mankind. Its impact ranges from a minor inconvenience requiring surgical caries removal and restorative treatment to excruciating pain and loss of masticatory function. While the role of plaque biofilm in caries causation is beyond refute, it is also becoming increasingly clear that strategies directed at eliminating specific caries-associated microorganisms, which are members of the endogenous microflora, have not only proven to be difficult but may also be unwise. The benefits of the use of topical fluorides in a wide variety of formulations and methods of delivery are universally accepted by the dental scientific and practicing community. This paper will focus on two commonly used approaches of fluoride delivery, namely, fluoride dentifrice and fluoride mouthwashes. Remineralization strategies other than fluoride will also be addressed, as well as the use of high fluoride preparations intended to arrest dental caries as part of the Atraumatic Restorative Treatment (ART) approach. The bulk of the paper will deal with fluoride dentifrice, which is probably the least appealing of these subjects, unless you include whitening ingredients, but it remains one of the most important caries prevention tools we have today.

## Dentifrices

Fluoride dentifrices have been shown in numerous clinical trials to be effective anticaries agents [1] and have been recognized as a major cause of the remarkable decline in caries prevalence in many developed countries [2,3]. Dentifrices have been widely adopted around the world as the principle means of delivering topical fluoride and obtaining caries preventive benefits. Over 95% of all dentifrices sold in the U.S. contain fluoride. While fluoride dentifrices have been extensively examined in several traditional narrative reviews, a recent systematic quantitative evaluation by Marinho *et al*. (2003)[4] (Cochrane Database of Systematic Reviews) provides the best evidence for the effectiveness of fluoride dentifrice. Based on a meta-analysis of 70 trials on the effectiveness of fluoride dentifrice compared to placebo for the prevention of dental caries in children, they found clear evidence that the use of fluoride dentifrices has a caries-inhibiting effect (average reduction in DMFS of 24%) on permanent dentition. Furthermore, the effectiveness of fluoride dentifrice may be relatively greater in individuals with higher caries experience, with increased fluoride concentration, increased frequency of use, and with supervised brushing. There was no evidence that the effect was dependent on background exposure to fluoridated water. The review provided little information on the effect of fluoride toothpaste on caries incidence in deciduous dentition. Similar overall conclusions were reached by another systematic review by Twetman *et al*. (2003) [5] as well as in a review conducted by Fluoride Recommendations Work Group for the Center for Disease Control and Prevention (2001)[6].

### Regulation of Fluoride Dentifrices

In the United States, fluoride dentifrice products are regulated as an over-the-counter (OTC) drug. After a process extending over 25 years, the Federal Drug Administration (FDA) issued on October 6, 1995, in the Federal Register (21 CFR Parts 310, 355, and 369), the final monograph "Anticaries Drug Products for Over-the-Counter Human Use" as part of its ongoing review of OTC drug products [7]. This monograph established the conditions under which OTC anticaries drug products are generally recognized as safe and effective. There was a one-year period when the document was open for review and comment, after which it became the law, or the final rule. The deadline for compliance with the monograph was later extended for one more year to give industry more time to react to the monograph and test its fluoride dentifrice products. The approval of new products with formulations that are outside the monograph requires a New Drug Application (NDA), which involves two clinical caries trials to establish effectiveness and safety.

To comply with the anticaries monograph on fluoride dentifrice products, all OTC fluoride dentifrice products must meet or exceed the soluble fluoride ion (F^- ^and PO_3_F^=^) level specified in the monograph for each fluoride compound (Table 1) and meet the test requirements for equivalence against the appropriate United States Pharmacopeia (USP.) fluoride reference standard (Table 2) for the animal caries reduction test, and either the enamel solubility reduction test or the fluoride enamel uptake test. The monograph refers to the Biological Testing Procedures for Fluoride Dentifrices (Federal Register Docket No. 80N-0042) for details on the accepted methods. The Standard for Fluoride Dentifrices, dated March 11, 1978, which describes the Laboratory Testing Profiles (LTPs), was developed by the Proprietary Association Subgroup on Fluoride Dentifrices. Several manufacturers have exercised the provision in the anticaries monograph for petitioning the FDA for approval to conduct alternative testing using intraoral appliance (IOA) models for compliance with the biological (animal) testing requirement. In 2001, the FDA announced a request for information and comments on the use of IOA (in situ) models as a substitute for the animal caries reduction test, with a deadline of January 14, 2002 (Federal Register/Vol. 66, No. 199) [8] The deadline was later extended to July 12, 2002, at the request of the Anticaries Task Group of the Consumer Healthcare Products Association. The anticaries monograph trail ends here.

**Table 1:** Concentration and Dosage of Anticaries Active Dentifrice/Rinse/Gel Ingredients Federal Register 21 CFR 355.10

**Sodium fluoride**	
Dentifrices	Dentifrices containing 850 to 1,150 ppm theoretical total fluorine in a gel or paste dosage form. Sodium fluoride 0.188 to 0.254% with an available fluoride ion concentration = 650 ppm.
Powders	Dentifrices containing 850 to 1,150 ppm theoretical total fluorine in a powdered dosage form: Sodium fluoride 0.188 to 0.254% with an available fluoride ion concentration of = 850 ppm for products containing the abrasive sodium bicarbonate and a poured-bulk density of 1.0 to 1.2 g/ml.
Treatment rinses	An aqueous solution of acidulated phosphate fluoride derived from sodium fluoride acidulated with a mixture of sodium phosphate, monobasic, and phosphoric acid to a level of 0.1 molar phosphate ion and which yields an effective fluoride ion concentration of 0.02%.
	An aqueous solution of acidulated phosphate fluoride derived from sodium fluoride acidulated with a mixture of sodium phosphate, dibasic, and phosphoric acid to a pH of 3.5 and which yields an effective fluoride ion concentration of 0.01%.
	Sodium fluoride 0.02% aqueous solution with a pH of approximately 7.
	Sodium fluoride 0.05% aqueous solution with a pH of approximately 7.
	Sodium fluoride concentrate containing adequate directions for mixing with water before using to result in a 0.02% or 0.05% aqueous solution with a pH of approximately 7.
**Sodium monofluorophosphate**	
Dentifrices	Dentifrices containing 850 to 1,150 ppm theoretical total fluorine in a gel or paste dosage form. Sodium monofluorophosphate 0.654 to 0.884% with an available fluoride ion concentration (consisting of PO_3_F^= ^and F^- ^combined) = 800 ppm.
	Dentifrices containing 1,500 ppm theoretical total fluorine in a gel or paste dosage form. Sodium monofluorophosphate 1.153% with an available fluoride ion concentration = 1,275 ppm.
**Stannous fluoride**	
Dentifrices	Dentifrices containing 850 to 1,150 ppm theoretical total fluorine in a gel or paste dosage form.
	Stannous fluoride 0.351 to 0.474% with an available fluoride ion concentration = 700 ppm for products containing abrasives other than calcium pyrophosphate.
	Stannous fluoride 0.351 to 0.474% with an available fluoride ion concentration = 290 ppm for products containing the abrasive calcium pyrophosphate.
Preventive treatment gel	Stannous fluoride 0.4% in an anhydrous glycerin gel, made from anhydrous glycerin and the addition of suitable thickening agents to adjust viscosity.
Treatment rinse	Stannous fluoride concentrate marketed in a stable form and containing adequate directions for mixing with water immediately before using to result in a 0.1% aqueous solution.

**Table 2:** U.S.P. Fluoride Dentifrice Reference Standards

Sodium Fluoride/Calcium Pyrophosphate (high beta-phase) (discontinued)
Sodium Fluoride/Silica
Sodium Fluoride/Sodium Bicarbonate
Sodium Monofluorophosphate/Calcium Carbonate
Sodium Monofluorophosphate/Dicalcium Phosphate
Sodium Monofluorophosphate (1000 ppm F)/Silica
Sodium Monofluorophosphate (1,500 ppm F)/Silica
Stannous Fluoride/Silica

### American Dental Association Evaluation Program

The ADA, through its Acceptance Program, provides another level of assurance to dental professionals and the public that fluoride dentifrice products are safe and effective. Many fluoride dentifrice products are submitted to the ADA Acceptance Program and, if they meet the required specifications, then obtain the ADA Seal of Acceptance as a symbol of a dental product's safety and effectiveness. Currently there are 47 fluoride dentifrice products from 12 different manufacturers with the ADA Seal. The current guidelines (as of May 1998) require clinical caries trials for new dentifrice formulations with substantial differences in chemical composition, such as a new fluoride source or a new abrasive system. Dentifrice formulations that are identical or similar in chemical composition to previously ADA-accepted products need to be supported by data for the following: total fluoride in fresh and aged samples; available fluoride in fresh and aged samples; one-minute fluoride release rate; and bioavailability in enamel. Like the FDA, the ADA allows some leeway in the type of testing that can be done. The ADA requirements are similar to the FDA requirements with the following exceptions: the one-minute fluoride release data is not required by the FDA; and caries testing in rats is not required by the ADA, although they accept this type of data. Another important difference is that the ADA requires companies to submit data for review by ADA Division Science staff and then to receive formal approval by the ADA Council on Scientific Affairs before giving the ADA Seal, while the FDA reserves the right to conduct unannounced regulatory audits to determine if companies are in compliance with the anticaries monograph. Also the ADA conducts internal laboratory testing on all fluoride dentifrice products requesting the ADA Seal to determine available fluoride and one-minute fluoride release.

### Impact of Regulatory and Marketplace Forces

There are some factors in the current environment that are not necessarily conducive to having the most effective products on the market. First, it is not in the FDA's regulatory purview to assure that OTC drug products are as clinically effective as possible, only that they are clinically effective and safe. The FDA is not necessarily interested in the superiority of one product over another unless a company is making unsubstantiated claims.

Second, in the current consumer-driven marketplace, dentifrice manufacturers have more of an incentive to pursue non-therapeutic cosmetic claims than to develop new classes of products. The only new clinically tested formulation approved by the FDA in recent memory has been Colgate Total, approved in 1996. Marketing yields a much higher return on investment than research and development for new products in the short term, which has shifted the emphasis away from anticaries effectiveness and toward cosmetic claims. As mentioned above, the approval of new products requires a NDA. The cost of running two- to three-year clinical caries trials has been a major deterrent to the development of new fluoride dentifrice formulations. The rising cost has been attributed to the need for a larger sample size and to the costs associated with participant accrual, higher regulatory and methodological standards, and higher infrastructure costs [9]. There is currently a high level of interest in the development and validation of models that include the early detection of non-cavitated lesions using visual- and technology based methods with the intent of shortening the length and cost of caries trials [10].

Third, over time the link between the anticaries monograph and actual clinical effectiveness is becoming weaker and weaker. Many of the clinical studies that were used as the basis for establishing the anticaries monograph were conducted well over 25 years ago. Many of these studies would not hold up today under the modern requirements for the design and conduct of randomized controlled trials (RCT) as covered by the CONSORT (Consolidated Standards of Reporting Trials) statement. We are now in a situation where very few of the currently marketed dentifrice products (Crest Cavity Protection Toothpaste, Colgate Total, and Colgate Cavity Protection) have ever been tested in clinical caries trials. All other products are currently being marketed based on meeting the requirements of the FDA anticaries monograph. Manufacturers are continually modifying formulations to make new marketing claims, due to changes in availability of ingredients and for cost considerations. While we can have some measure of confidence that the required in vitro and animal testing provide assurance of the efficacy of dentifrice products, there must also be a note of caution that we are moving farther and farther away from the actual clinical evidence of the effectiveness of fluoride products.

Fourth, the testing requirements of the anticaries monograph have a number of limitations. Many of the LTPs involve antiquated methods that date back to the 1970s or earlier and don't reflect our current understanding of the mechanism of action of fluoride. There is a disconnect between what is considered by most experts in the field to be the main mechanism of action of fluoride--the ability to enhance remineralization--and the monograph testing requirements. The enamel solubility reduction testing is not highly regarded as an appropriate methodology, yet this test is in part the basis for the marketing of many products. Some of the methods require reagents that are no longer available. The regulations are very rigid, and any modification of existing methods or use of new methods requires a citizens' petition, which can be an expensive and time-consuming process. This in effect stifles the development of newer testing methods. Most of the LTPs have not been adequately validated based on their ability to demonstrate a fluoride dose response, which is a requirement that has been established for *in situ *demin/remin models [11].

Lastly, the requirements for statistical analysis are not well defined. This point is recognized by the FDA, and statistical methods were included in their request for information and comments on the use of intraoral appliance models (mentioned above).

To address these problems, future laboratory methods should be based on performance criteria and not specific cookbook methods. Model systems should be required to: exhibit a fluoride dose response (0, 250, 550, 1100 ppm F); run internal controls for all tests (0, 250, gold standard); have sample size sufficient to achieve statistical power for demonstrating equivalence; and use defined statistical methodology to establish equivalency ("as good as") with an appropriate clinical gold standard.

### Dentifrice Ingredients

The formulation of modern therapeutic fluoride dentifrices has evolved into a sophisticated art and science. Most fluoride dentifrice products contain an active fluoride compound, water, abrasive system, surfactants, binding agents, humectants, flavoring and sweetening agents, coloring, and preservatives. In the last 20 years, dentifrices have been increasingly used as a vehicle for other additives including calculus inhibiting agents (tartar control), anti-plaque/anti-gingivitis agents, tooth desensitizing agents, anti-oral malodor/breath freshening agents, whitening/stain removal systems, and remineralizing agents. The author's opinion, which may be somewhat controversial, is that the only claim that has been associated with a long-term health benefit is the anticaries effects of fluoride, and thus this claim must be given precedence above all others.

It has long been recognized that the effectiveness of fluoride in dentifrice products is greatly influenced by the compatibility of fluoride with other ingredients. The realization of this problem dates back to the first clinical trials testing fluoride dentifrice reported by Bibby (1945)[12] in which the incompatibility of NaF with the abrasive (dicalcium phosphate) compromised the effectiveness of the formulation. The first dentifrice formulation shown to be clinically effective was reported by Muhler *et al*. (1954)[13], combining SnF_2 _with a calcium pyrophosphate abrasive system, which led to the launching of Crest in 1956. This early formulation had a limited shelf life due to interaction between the fluoride compound and the abrasive, and was later replaced by the modern NaF/silica combination.

Three sources of fluoride are covered by the FDA anticaries monograph, namely, stannous fluoride (SnF_2_), sodium monofluorophosphate (Na_2_PO_3_F), and sodium fluoride (NaF) (Table 1). Due to the high level of commercial interest, it is very difficult to establish a clear scientific position on the relative effectiveness of these agents from reading the published literature. There have been several reviews on the relative merits of NaF vs. MPF, with opposing sides of the issue reaching different conclusions from basically the same studies [14-19]. While all three fluoride compounds have been proven to be clinically effective on purely theoretical grounds, NaF, when delivered in an appropriately formulated dentifrice, appears to deliver fluoride in a form that is the most congruent with the current understanding of the mechanism of action of fluoride [20]. Amine fluoride dentifrices marketed in Europe have also been shown to be clinically effective, but are not approved for use in the US.

Table 1 lists concentration and dosage form of the active ingredients covered by the FDA OTC anticaries monograph. All dentifrices currently marketed in the US are formulated to contain either 1000 or 1100 ppm F, mostly in the form of NaF and MFP. There is evidence of an improved anticaries effect with higher F concentrations for both MFP and NaF based on various clinical studies that have used different formulations, examiners, and populations [21]. A higher concentration MFP dentifrice product (Extra Strength Aim) with 1500 ppm F was approved through the FDA's NDA process with the requirement that the labeling state "For dentifrice products containing 1,500 ppm theoretical total fluorine. Adults and children over 6 years of age may wish to use this extra-strength fluoride dentifrice if they reside in a nonfluoridated area or if they have a greater tendency to develop cavities." This product is no longer being marketed, and currently there aren't any extra-strength OTC fluoride dentifrice products being sold in the US. Interestingly, in Europe most of fluoride dentifrice products have 1,500 ppm F where they are regulated by the European Union as cosmetic products.

Abrasive systems are included in dentifrices to control the buildup of stain on teeth that naturally occurs in most individuals. Commonly used dentifrice abrasives included calcium carbonate (chalk), dicalcium phoshphate, silica, or sodium bicarbonate. Abrasives are well-known to play a very important role in the availability and rate of release of fluoride. As many individuals brush their teeth for less than one minute, it is critical that the fluoride is released from the dentifrice within that time frame to be clinically effective. The FDA anticaries monograph does not require this particular test (ADA does), but relies on the biological test methods (rat caries and/or enamel solubility reduction, fluoride uptake) as proof that fluoride is available. The other ingredients in dentifrice products can also impact fluoride effectiveness. Sodium lauryl sulphate is the most commonly used anionic surfactant in dentifrices and is known to have a slight antiplaque effect, but may also interfere with fluoride uptake by enamel. Dentifrice products with strong taste characteristics may cause excessive salivary stimulation, which would increase the rate of fluoride clearance from the mouth.

Concerns have been raised that agents that inhibit calculus formation, such as pyrophosphate, may also interfere with the remineralization process. This contention has been countered by studies which have indicated that the addition of pyrophosphate to a fluoride dentifrice does not interfere with the anticaries effects of fluoride, mainly based on in vitro and animal studies [22]. While tartar control fluoride dentifrice products have been shown to be effective in clinical caries trials, there is no definitive clinical evidence that they are equivalent to regular fluoride dentifrice. An interesting counterpoint is that marketing claims may encourage the increased use of fluoride dentifrice products. For example, even if a dentifrice additive, such as a whitening agent, reduces the effectiveness of a fluoride dentifrice compared to a regular dentifrice product, the desire to have whiter teeth may result in increased frequency and length of tooth brushing, and this could offset a potentially negative effect. Dentifrice products that provide maximum caries protection and are attractive to consumers would be ideal.

### Factors Affecting Dentifrice Effectiveness

In addition to the inherent properties of a fluoride dentifrice product, biological and behavioral factors can modify its anticaries effectiveness. All of these factors interplay in what can be described as the "application" phase (the initial interaction of relatively high concentrations of fluoride with the tooth surface and plaque), and the "retention" phase (the fluoride remaining in the mouth after brushing that is retained in saliva, plaque and plaque fluid, the tooth surface, and oral soft tissue reservoirs) [23-25]. Behavioral factors include the frequency of dentifrice use, length of brushing, rinsing practices after brushing, the time of day that dentifrice is applied, and amount of dentifrice applied to the brush. It is well established that the frequency of use has a major influence on effectiveness. Bushing twice per day or more has a greater preventive effect than once per day [4]. Length of the brushing time (application phase) determines how long the relatively high fluoride concentration in the dentifrice slurry stays in contact with the teeth and plaque, allowing fluoride uptake to take place. The higher the fluoride concentration, the greater the driving force for fluoride diffusion through plaque toward the tooth surface [25]. Rinsing behaviors after toothbrushing affect the amount of fluoride retained in the mouth [23,25-27] and have been reported to affect caries experience [28,29]. Physiologic (biological) factors, mainly salivary flow rate during and after fluoride application influence the rate of fluoride clearance [30][31]. Bedtime use of fluoride dentifrice results in longer fluoride retention than daytime application due to greatly decrease salivary flow during sleep [23,25]. The amount of fluoride applied to the toothbrush (dose) is not as important as the concentration of available fluoride in a dentifrice. Reduced fluoride concentration dentifrices are not as effective as regular concentration products [32,33]. The fluoride dose is, however, important in regard to enamel fluorosis in children under six years of age because of dentifrice ingestion. For this reason, reducing the amount of fluoride applied is a better strategy than lowering the dose of products intended for use by children (discussed below).

### Safety

While dentifrice products have a long history of safety, there is an ongoing concern associated with dental fluorosis due to fluoride ingestion in children under age six [34]. Studies have shown that for children 1–3 years, 30–75% of the dentifrice is ingested, and for children 4–7 years 14–48% is ingested [35]. As with any OTC drug product, precautions need to be taken to prevent overdose. The FDA requires labeling of all fluoride dentifrice products to include a statement "to minimize swallowing use a pea-size amount in children under six." Making childproof caps available on fluoride dentifrice products intended for use by children has been recommended. Another approach would be to provide metered dentifrice delivery systems for children under age six, which could be set to dispense the correct amount of fluoride depending on the body weight of the child.

### Summary

Market forces and the current regulatory and professional environment do not appear to favor developing dentifrices with improved efficacy. The oral care market has grown substantially in the last few years. Annual sales are currently over the 7 billion dollar mark and are projected to be $8.5 billion in 2007, mainly due to consumer interest in whitening products. While consumer preferences are tightly held industrial secrets, it is safe to say that consumers believe that all toothpastes with fluoride provide protection and their choices are driven by other factors, such as taste, breath freshening, or specific claims, such as whitening or tartar control, with preferences varying depending on age group.

One way of addressing oral health disparities is to make certain that at-risk individuals are obtaining the maximum benefit from fluoride dentifrice by making available the most effective products and educating individuals on how to obtain the greatest benefit. Clearly there needs to be a balance between regulatory oversight and free market economy. If the rules and regulations are too stringent or impossible to attain, more effective anticaries products may never reach the marketplace, and the public will never receive the benefit of these products. On the other hand, some form of active market surveillance may be warranted. A recent study has reported on the quality control of dentifrices from non-established market economy countries [36]. Deficiencies were found in the total and free ionizable fluoride concentration in a random selection of 101 dentifrices. Products with deficient fluoride availability may be making their way into this country. Dentifrices made in Mexico, which likely have not been tested according to FDA regulatory standards, can be easily purchased in many Mexican grocery stores in the United States.

## Mouthrinses

The use of mouthrinses to deliver chemotherapeutic agents is well accepted by the public, both by self administration [37] and under supervision, mainly in school fluoride rinsing programs [38]. Mouthrinse formulations are generally much simpler than dentifrices, and compatibility problems are not as large an issue as they are with dentifrice products. While mouthrinses are a heavily utilized oral care vehicle with over 120 million mouthwash users in the US, fluoride mouthrinses represent only 7% of the total mouthrinse business in the US, and thus, there is considerable room for increasing use of this approach for delivering fluoride.

Many types of mouthrinse active ingredients have been evaluated for their plaque- reducing effectiveness and ability to reduce mutans streptococci, including chlorhexidine, essential oils, triclosan, cetylpyridinium chloride, sanquinarin, sodium dodecyl sulphate, and various metal ions (tin, zinc, copper). However, the evidence supporting the effectiveness of antiplaque agents in preventing dental caries, with the possible exception of chlorhexidine, is very limited; therefore, fluoride-containing mouthrinse will be the main focus here. A meta-analysis of 34 studies by Marinho *et al*., 2003 [39] (Cochrane Database of Systematic Reviews) reported that the supervised use of fluoride mouthrinse by children is associated with a clear reduction (preventive fraction of 26%) in caries increment. Both daily rinsing with 0.05% NaF (226 ppm F) and once a week/once every two weeks rinsing programs with 0.2% NaF (900 ppm F) were found to be effective. While the reviewers could find no evidence that the effect was dependent on baseline caries level, background fluoride exposure, or mouthrinsing frequency, they cautioned that there was limited power to detect such relationships. Twetman *et al*. (2004) [40] in their systematic review of non-selected populations of various age groups found a preventive fraction of 29% for daily and weekly rinses in the permanent teeth of schoolchildren and adolescents with no additional fluoride exposure. Several authors have questioned if fluoride mouthrinsing is a cost-effective strategy on a population basis and recommended that its use be targeted to high-caries-risk individuals and groups [41,42]. Twetman *et al*. (2004) [40] also questioned the additional anticaries benefit of fluoride mouthrinses in children who regularly use fluoride dentifrice and recommended that future research is necessary to determine if fluoride mouthrinse is effective in high-caries-risk and caries-active individuals.

From a mechanistic perspective, fluoride mouthrinses can lead to higher levels of oral fluoride retention than fluoride dentifrice, depending on behavioral practices after toothbrushing. Zero *et al*. (1992) [25] reported that salivary fluoride retention, after fluoride mouthrinse (226 ppm F) use was significantly greater than after brushing with fluoride dentifrice (1100 ppm F), based on integrated F values over the first two hours after application. The common practice of rinsing with tap water after toothbrushing greatly reduced oral fluoride retention. This finding suggested that the combination of brushing with fluoride dentifrice followed by fluoride mouthrinse use may be beneficial.

### Regulation of Mouthrinses

The FDA classifies mouthrinses as either cosmetic or therapeutic, or a combination of the two. The cosmetic mouthrinses are over-the-counter products that are mainly intended as mouth fresheners. Therapeutic rinses can be sold as prescription or over-the-counter products that have an added active ingredient and are marketed as antiplaque/antigingivitis drug products and anticaries drug products. Table 1 lists the formulations of fluoride mouthrinses that are included in the FDA anticaries monograph. The monograph does not require any additional laboratory testing for fluoride mouthrinse. Due to concerns about fluoride ingestion, mouthrinsing is not recommended for children under six years of age.

## Remineralizing agents

Recently there has been broader recognition that the appropriate management of dental caries should involve "improved diagnosis of early non-cavitated lesions and treatment for prevention and arrest of such lesions," based on a statement from the Consensus Development Conference on the Diagnosis and Management of Dental Caries Throughout Life, sponsored by the National Institutes of Health, 2001 [43]. Furthermore, the current interest in biomimetics and regenerative biology at NIDCR opens up new opportunities for a long-held interest in remineralization or repair of caries lesions without using traditional dental materials. Claims that the destructive effects of the caries process can be arrested and possibly reversed date back to the turn of the 20^th ^century (reviewed by Koulourides, 1986; Kashket, 1999)[44,45]. Evidence supporting the reversal (remineralization) of early lesions has come mainly from *in vitro *and *in situ *studies of partially demineralized enamel and dentine, and to a lesser extent from direct clinical trials [46]. However, based on a systematic review, Bader *et al*. (2001) [47] concluded that the evidence was insufficient to establish the efficacy of methods (mainly involving the use of fluoride) for arresting or reversing progression of early lesions because of a limited number of studies and lack of adequate statistical testing.

Ideally, remineralizing agents need to rapidly precipitate on partially demineralized tooth structure and transform into a more stable, less acid-soluble apatite than the hard tissue replaced. They would need to do this in the presence of saliva and before the next acid challenge comes in contact with the newly precipitated mineral. If the mineral phase that is formed is soluble in saliva or under acidic conditions, it will be rapidly lost. On the other hand, mineral that is taken up by the enamel may serve as a reservoir that could be released into fluid phase surrounding the enamel crystals during a caries attack and serve as a substrate for subsequent remineralization. If complete remineralization of subsurface (white spot) lesions is the goal, then the agent must also be able to diffuse past the pellicle-covered enamel surface and into the subsurface lesion area. It has been found to be very difficult to diffuse calcium and phosphate ions into the deeper layers of carious enamel, and most remineralization is confined to the surface of a carious lesion [48]. Under conditions that favor remineralization, calcium will rapidly adsorb onto the surface layer and precipitate in the pores, thus blocking access to the deeper subsurface enamel lesion. *In vitro *studies have shown that the penetration of calcium and phosphate can be enhanced if proteins (pellicle and enamel proteins) are removed from the enamel surface layer using either sodium hypochloride [49] or acid etching [50]. There is also some evidence that acidulated calcifying fluids enhance the degree of remineralization. Flaitz and Hicks (1994) [50] reported that an acidulated calcifying fluid (pH 5.0) was more effective than a neutral calcifying fluid (pH 7.0) in enhancing remineralization. The calcium concentration was also found to be important. Calcifying fluid with 1 mM calcium resulted in more remineralization of the entire lesion than calcifying fluid with 3 mM calcium, while the higher concentration calcifying fluid resulted in a greater increase in the surface zone depth.

### Remineralizing strategies

There are a number of approaches that have been considered to enhance the natural repair process by calcium and phosphate in saliva and plaque fluid. These include: 1) combining remineralizing agents with fluoride to enhance fluoride's anticaries effectiveness; 2) combining remineralizing agents with a lower dosage of fluoride to decrease the possibility of dental fluorosis in young children without losing effectiveness; and 3) the use of remineralizing materials as independent agents with fluoride use in the background. Delivery methods for remineralization materials include toothpastes, mouthrinses, gels, pastes, chewing gums, lozenges, and foods and beverages.

Fluoride remains the best established remineralization strategy, although definite evidence that it can clinically reverse early caries lesions remains limited as noted above. Trace amounts of fluoride in saliva are effective in shifting the balance from demineralization to remineralization. This is attributed to the fluoride-enhanced precipitation of calcium phosphates, and the formation of fluorhydroxyapatite in the dental tissues [51,52]. It has been proposed that the formation of calcium fluoride-like material in plaque and on the surface of enamel may serve as a reservoir for both calcium and fluoride. The challenge has been to keep the calcium separate from the fluoride until they can interact with the tooth surface. This is the basis of the strategy developed by Chow and Takagi (1991) [53], which uses a two-solution system, one containing sodium fluorosilicate and the other calcium chloride, which will be discussed below.

Many compounds have been identified that are capable of delivering calcium and phosphate to the oral cavity. Highly soluble compounds are capable of delivering high concentrations; however, they can be rapidly cleared from the mouth unless they precipitate in plaque or on the tooth surface. Highly insoluble compounds are considered to be of limited value, unless they become trapped in plaque and can be hydrolyzed by plaque enzymes and/or can be dissolved under low plaque pH conditions. The most tested compounds include calcium glycerophosphate and calcium lactate (Tables 3 and 4, respectively). Dicalcium phosphate dihydrate (DCPD), which has a lower solubility, has been studied in chewing gum clinical caries trials and in dentifrice studies (Table 5). Calcium carbonate is another insoluble compound that is used as a dentifrice abrasive. Tenovuo *et al*. (1997) [54] evaluated salivary calcium levels after human subjects used a lozenge that contained calcium carbonate. Calcium levels were found to be elevated for only the first 2–4 minutes after subjects sucked the lozenge.

**Table 3:** Calcium Glycerophosphate

Model System Reference	Delivery System	Conc. Tested	Caries Inhibition	Demin Inhibition	Remin Enhancement	Plaque Enrichment
Human Children						
Brook *et al*. (1975) [73]	tablet 4X/day for 84 days	1%				P ++ Ca +
Duke *et al*. (1979) [74]	CaCO_3_/MFP dentifrice	0.13%				P ++ Ca ++
Naylor and Glass (1979) [75]	CaCO_3_/MFP dentifrice	0.13%	+/-			
Mainwaring and Naylor (1983) [76]	CaCO_3_/MFP dentifrice	0.13%	+			
						
Human Adult						
Sidi (1989) [77] 1 hr after brushing	CaCO_3_/MFP dentifrice	0.13%				P + Ca ++
Sidi & Wilson (1991)[78] 24 hr after brushing	CaCO_3_/MFP dentifrice	0.13%				most effect lost after 24 hr

**Table 4:** Calcium Lactate

Model System Reference	Delivery System	Conc. Tested	Caries Inhibition	Demin Inhibition	Remin Enhancement	Plaque Enrichment
Human Adult						
van der Hoeven *et al*. (1989) [79]	rinse	0.165 M				P ++ Ca ++
Schaeken & van der Hoeven (1990) [80]	rinse	5%				P ++ Ca +++ [d1]
						
*In situ *Caries						
Brudevold *et al*. (1985) [81]	10% Sucrose (S) rinse	5% (0.162 M)		++		
Kasket & Yaskell (1997) [82]	10% S rinse	0.05 M 0.1 M 0.15 M		++ ++ +++ [d2]		

**Table 5:** Dicalcium Phosphate Dihydrate

Model System Reference	Delivery System	Conc. Tested	Caries Inhibition	Demin Inhibition	Remin Enhancement	Saliva/Plaque Enrichment
Human Children						
Averill & Bibby (1964)[83]	flour and sugar	2.0%	-			
Finn & Jamison (1967)[84]	sugar chewing gum	10%	+			
Richardson *et al*. (1972)[85]	sugar chewing gum	10%	+			
DePaola (1993) [86]	MFP dentifrice	49%	+			
Boneta *et al*. (2001)[87]	NaF dentifrice/dual chamber	48%	++			
Silva *et al*. (2001)[88]	NaF dentifrice/dual chamber	48%	+			
						
*In situ *Remin						
Zhang *et al*. (1995)[89]	MFP dentifrice	49%			++	Ca ++
Sullivan *et al*. (2001)[90]	NaF dentifrice/dual chamber	48%			+	Ca ++
Itthagarun *et al*. (2005)[91]	sugar free gum	2%			++	

Several newer compounds are also under consideration as remineralizing agents. Casein phosphopeptide (CPP) amorphous calcium-phosphate (ACP) complexes have been reported to increase the level of calcium phosphate in plaque [57], to inhibit enamel *in situ *demineralization [55], to enhance *in situ *remineralization [56,57], and to reduce caries activity in the rat [58]. CPP-ACP nanocomplexes are thought to provide a reservoir of calcium and phosphate ions to maintain a state of super saturation with respect to tooth enamel and buffer plaque pH, and to provide ions for tooth enamel remineralization.

A number of calcium phosphate compounds have been evaluated by researchers at the ADA Health Foundation, Paffenbarger Research Center. Chow *et al*. (1994) [59] compared the effects of two experimental chewing gums containing calcium phosphates on whole saliva. The gums contained either 5% monocalcium phosphate monohydrate (MCPM) or an equimolar mixture of tetracalcium phosphate with dicalcium phosphate anhydrous (TTCP-DCPA). The MCPM gum significantly increased salivary calcium concentration compared to a control gum for the first 12 minutes of the 16-minute test period, while the TTCP-DCPA gum produced significant increases in calcium for the entire test period. The calcium concentrations produced by the TTCP-DCPA gum were also significantly higher than the MCPM gum between the 6- and 16-minute sampling points. The degree of supersaturation with respect to hydroxyapatite was significantly increased by both gums compared to control gum. The degree of supersaturation in the samples from the TTCP-DCPA gum treatment was also significantly greater than the corresponding samples from the MCPM gum treatment, suggesting that the TTCP-DCPA gum may have greater remineralization potential. A chewing gum containing 2.5% α-tricalcium phosphate was also found to significantly increase the ion activity product of saliva with respect to hydroxyapatite, suggesting an increase in remineralizing potential compared to a control gum [60]. Two solution fluoride rinse involving sodium hexafluorosilicate and calcium chloride has been shown to deposit large amounts of calcium fluoride-like material on the enamel surface [53] and to produce greater *in situ *remineralization than a fluoride rinse with twice the fluoride concentration [61]. Amorphous Calcium Phosphate compounds (ACPs) are considered prime candidates for remineralization therapy due to their high solubility under oral conditions and ability to rapidly hydrolyze to form apatite [62]. This technology has been applied in the form of a dentifrice (Enamelon) with a partitioned delivery system to separate the active ingredients NaF and phosphate on one side and calcium component on the other side of the tube. The different studies conducted to support the clinical efficacy of this partitioned dentifrice have been systematically reviewed by Clarkson and Rafter (2001) [63]. These reviewers suggested that the technology has promise based on several animal and *in vitro *studies. The one human clinical trial, on a small number of subjects who had received head and neck radiation [64], failed to show an improvement in preventing coronal caries when using the partitioned dentifrice compared to a conventional NaF dentifrice, but it was found to be better than the conventional fluoride dentifrice in preventing root caries.

Other investigators have combined fluoride with other ingredients intended to maximize the remineralizing process. Featherstone *et al*. (1982) [65] evaluated a remineralizing solution consisting of 2 mM calcium (calcium nitrate), 0.1 mM zinc (zinc chloride), 0.1 mM strontium (strontium chloride), 10 mM tartaric acid, 0.3 mM potassium phosphate, and 0.6 mM sodium fluoride (pH 6.0). This combination was reported to be effective in rapidly remineralizing artificial carious lesions *in situ*. However, this finding was not confirmed by a clinical study involving remineralization of incipient interproximal lesions [66]. These authors considered the discrepancy with Featherstone's findings to be due to difficulty of diffusing minerals through the thicker plaque generally found in interproximal areas. Pearce and Nelson (1988) [67] tested a solution consisting of 500 mM urea, 20 mM calcium chloride, 12 mM sodium phosphate, 4.72 mM monofluorophosphate, and 0.28 mM fluoride (pH 5.0). They reported increased remineralization efficacy compared to a fluoride dentifrice using an *in situ *caries model.

### Challenges

There are several challenges to establishing the clinical effectiveness of remineralization agents: 1) They must demonstrate a benefit over and above an established and highly effective agent, namely, fluoride. 2) They must provide a remineralizing benefit in addition to the natural remineralizing properties of saliva. Under most physiological conditions, the concentrations of calcium and phosphate in saliva and plaque fluid are supersaturated with respect to enamel and, thus, favor remineralization over demineralization. Individuals with decreased salivary flow, however, may benefit from mineral supplementation. 3) The organic constituents in saliva can serve as accelerators and inhibitors of the remineralization process. Teeth are covered by the acquired pellicle, which has been shown to retard remineralization. 4) If sugar-free chewing gum is the delivery vehicle, chewing gum has a major remineralizing effect in and of itself, which makes it more challenging to show an additional benefit when using gum as the delivery vehicle. 5) Too much of a good thing could possibly disrupt the mineralization homeostasis of the mouth and favor calculus formation.

## Caries arrestment agents

Different topical agents, such as silver nitrate, stannous fluoride, sodium fluoride, silver fluoride and silver diamine fluoride have been applied clinically at high concentrations with the intent of arresting active caries lesions and/or to prevent further caries progression [68-72]. Clinical protocols have included their use with and without prior removal of caries tissues, and with and without the subsequent placement of restorative material. Of greatest interest is the use of these agents in non-traditional approaches for treating caries lesions, commonly referred to as Atraumatic Restorative Treatment (ART). This approach typically involves minimal removal of caries tissues by excavation with hand instruments without local anesthesia and restoration with glass ionomer cement.

One agent in particular, silver diamine fluoride (SDF), has the best support for its effectiveness, based on a 30-month prospective controlled clinical trial reported by Chu *et al*. (2002) [72]. The study involved 376 preschool Chinese children with caries in their maxillary primary anterior teeth. Subjects were sequentially assigned to one of five treatment groups: excavation + 38% silver diamine fluoride [Ag(NH_3_)_2_F] applied every 12 months; SDF applied every 12 months; excavation + 5% NaF varnish applied every 3 months; 5% NaF varnish applied every 3 months; water control. They found that annual application of SDF was more effective in arresting dentin caries than application of fluoride varnish every 3 months. Furthermore, the removal of caries tissue did not improve the effectiveness of SDF or fluoride varnish to arrest dentin caries. The investigators did observe that in the case of the fluoride varnish only, caries excavation reduced the proportion of teeth that were blackened, and thus had an esthetic benefit. The apparent effectiveness of SDF in arresting dentin caries without the necessity of prior caries removal may have advantages in certain community oral health programs where access by trained dental professionals is limited or not available. This of course would need to be balanced against esthetic concerns; however, it was reported that this was not a cause for parental dissatisfaction in the Chu *et al*. study on a Chinese population.

## Conclusion

Most of the recent innovations in oral care products have been directed toward making cosmetic marketing claims. This has left a wide opening for innovation and cross pollination from other scientific fields to fully exploit our mechanistic understanding of dental caries and its prevention.

The dental profession needs to help find incentives for the oral health care industry to develop more effective products that give consumers what they need as well as what they want. We also need to raise the level of social consciousness to support the development of cost effective and culturally effective caries management strategies targeted at high caries risk individuals.

## Competing interests

The author declares that he has no competing interests.
